# Neglected intrahepatic foreign body following penetrating thoracoabdominal injury: a 10-month delayed presentation

**DOI:** 10.1093/jscr/rjag398

**Published:** 2026-07-11

**Authors:** Nicholas Wijaya, Hanif Ardiansyah Sulistya, Clarishella Melvina Deinera, Aditya Rachmat Febrianto, Chaidar Muttaqin

**Affiliations:** Emergency Department, Dr. Soepraoen Army Hospital, Jl. S. Supriadi No. 22, Sukun, Malang 65147, Indonesia; Emergency Department, Dr. Soepraoen Army Hospital, Jl. S. Supriadi No. 22, Sukun, Malang 65147, Indonesia; Center for Global Surgery, ARC Institute, Galaxy Bumi Permai B2-4, Sukolilo, Surabaya 60111, Indonesia; Emergency Department, Dr. Soepraoen Army Hospital, Jl. S. Supriadi No. 22, Sukun, Malang 65147, Indonesia; Department of General Surgery, Dr. Soepraoen Army Hospital, Jl. S. Supriadi No. 22, Sukun, Malang 65147, Indonesia; Department of Surgery, Faculty of Medicine, Brawijaya University, Dr. Saiful Anwar General Academic Hospital, Jl. Jaksa Agung Suprapto No. 2, Klojen, Malang 65112, Indonesia; Department of Surgery, Faculty of Medicine, Brawijaya University, Dr. Saiful Anwar General Academic Hospital, Jl. Jaksa Agung Suprapto No. 2, Klojen, Malang 65112, Indonesia; Department of Cardiothoracic and Vascular Surgery, Dr. Soepraoen Army Hospital, Jl. S. Supriadi No. 22, Sukun, Malang 65147, Indonesia

**Keywords:** intrahepatic foreign body, neglected foreign body, penetrating trauma, liver injury

## Abstract

Intrahepatic foreign body (FB) is a rare but clinically important condition that often presents late due to nonspecific symptoms and the absence of a clear history of penetration. We report a 20-year-old woman with a neglected intrahepatic FB after penetrating thoracoabdominal trauma, presenting after a 10-month delay with chest-dominant symptoms. Computed tomography revealed a hyperdense FB with a transcostal trajectory traversing the sixth intercostal space and penetrating hepatic segment III, associated with rib callus formation and a fistulous tract adjacent to the stomach. Exploratory laparotomy excluded gastrointestinal injury, and the FB was removed without complications. This case highlights an uncommon traumatic mechanism and an atypical thoracic presentation that masked underlying hepatic injury. Strict adherence to Advanced Trauma Life Support principles, particularly during the Exposure phase, and timely imaging are essential to prevent diagnostic delays that may lead to serious hepatic complications.

## Introduction

An intrahepatic foreign body (FB) is a rare but clinically important condition that often presents late due to nonspecific symptoms and the absence of a clear history of ingestion or penetrating injury, frequently after complications such as liver abscess or sepsis have developed [1, 2].

FBs may reach the liver via migration from the gastrointestinal (GI) tract following ingestion, the most common route, or through direct penetration of the abdominal or thoracic wall, hematogenous spread, or iatrogenic causes [3]. Sharp objects such as fish bones, sewing needles, metallic pins, and toothpicks may perforate the GI wall without acute symptoms and migrate into the hepatic parenchyma [1]. Without appropriate imaging, these cases are often misdiagnosed as cryptogenic liver abscess, resulting in retained FB and potentially fatal complications [2].

This report describes a neglected intrahepatic FB following penetrating thoracoabdominal trauma, presenting after a 10-month delay with an unusual thoracic–intercostal trajectory. The case highlights diagnostic challenges and emphasizes the importance of maintaining clinical suspicion. This case report is presented in accordance with the SCARE 2023 guidelines [4].

## Case presentation

A 20-year-old woman presented on 18 December 2025 with progressive left anterior chest pain (Visual Analogue Scale 6/10) and a palpable lower thoracic mass aggravated by movement and deep inspiration. She had sustained penetrating thoracoabdominal trauma involving glass fragments in a motorcycle accident 10 months earlier, leaving a keloid scar over the left lower chest wall. Further evaluation had been advised at a rural facility, but she did not pursue follow-up as symptoms were considered self-limiting.

**Figure 1:**
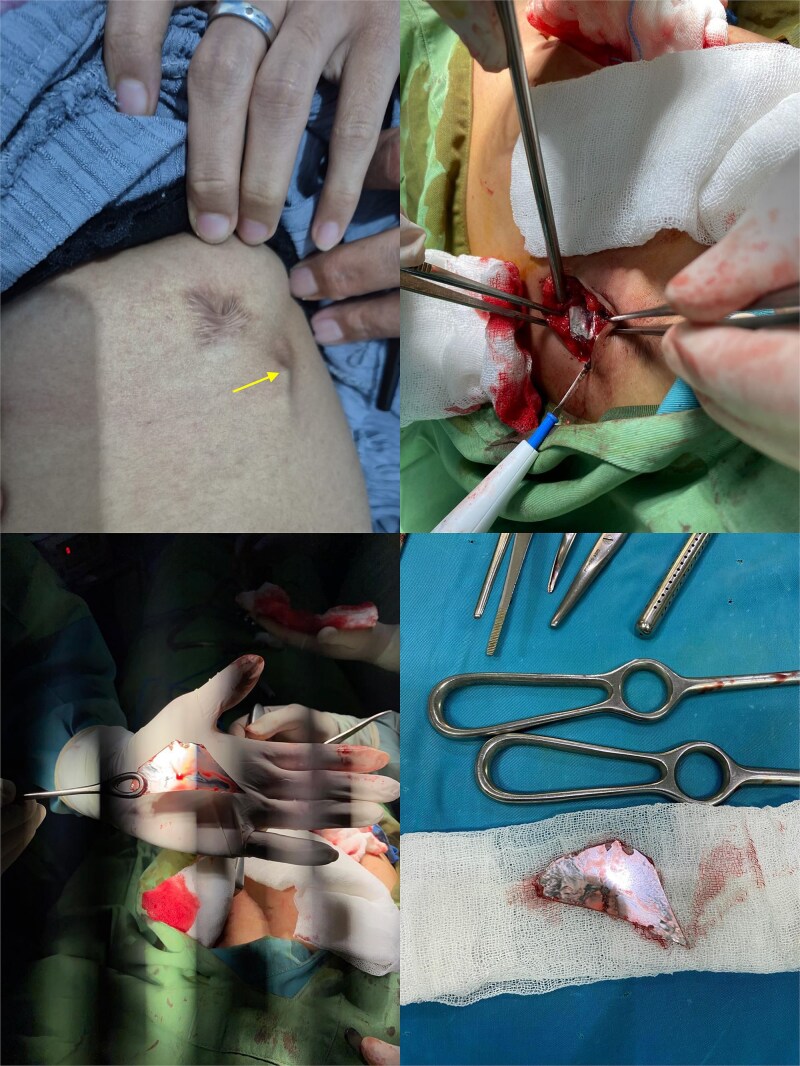
Picture 1 depicts palpable FB at scar site shown, indicated by an arrow; picture 2 depicts local incision superolateral to the previous scar site which revealed buried FB; pictures 3 and 4 depict the FB upon retrieval suspected of motorcycle mirror fragment.

On admission, she was alert (Glasgow Coma Scale E4V5M6) and hemodynamically stable, with a blood pressure of 100/60 mmHg, a heart rate of 80 beats per minute, a respiratory rate of 18 breaths per minute, and an oxygen saturation of 96% on room air. She measured 157 cm in height and weighed 48 kg (Body Mass Index 19.47 kg/m^2^). Physical examination revealed localized tenderness and a palpable mass over the left lower anterior chest wall, with a well-defined keloid scar measuring ~1.2 × 0.9 cm at the left sixth intercostal space (Fig. 1). No signs of peritonitis or acute abdominal pathology were present.

**Figure 2:**
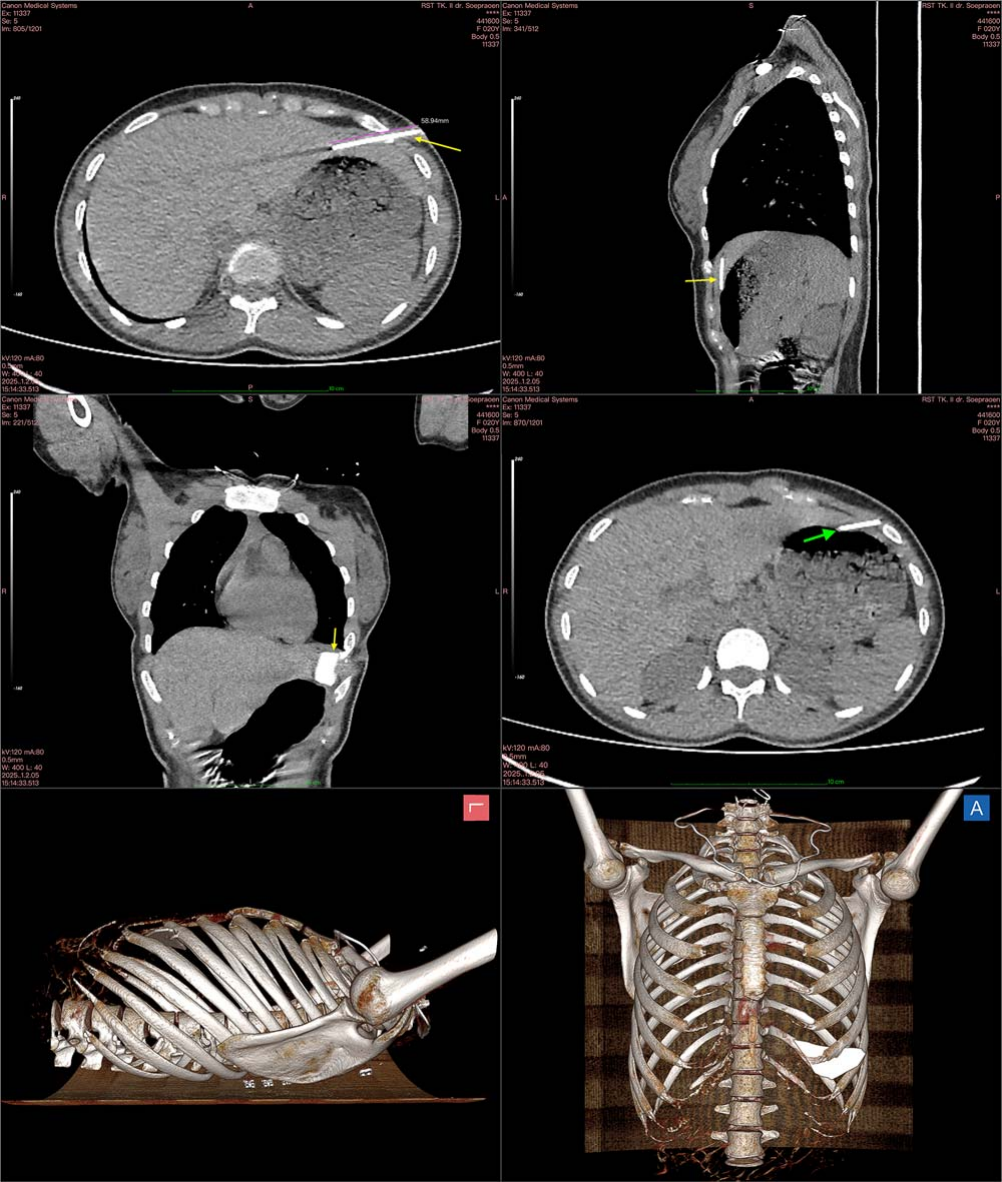
Chest CT with axial (top-left and middle-right), sagittal (top-right), coronal (middle-left) view, and 3D reconstruction with left (bottom-left) and anterior (bottom-right) view; FB is indicated by arrows and measured using a virtual ruler; an additional arrow identifies the suspected gastric perforation due to FB penetration (middle-right).

Computed tomography (CT) of the thorax and upper abdomen demonstrated a hyperdense FB measuring ~0.3 × 0.6 × 3.3 cm (≈1400 Hounsfield Unit), extending from the subcutaneous tissue of the left hemithorax, traversing the sixth intercostal space, and penetrating hepatic segment III to a depth of ~3.7 cm. Callus formation of the left sixth rib and a linear fistulous tract with the distal tip adjacent to the gastric wall were noted, raising concern for possible GI involvement. No free fluid, pneumoperitoneum, or active bleeding were identified (Fig. 2).

Laboratory investigations were within normal limits (Table 1). Exploratory laparotomy was performed on 19 December 2025 to exclude GI injury. However, no perforation was identified. The FB was removed through a localized incision superolateral to the keloid scar at the sixth intercostal space (Fig. 1). Intraoperatively, a fibrously encapsulated glass side-mirror fragment measuring ~7 × 4 cm was found penetrating hepatic segment III, associated with a small hepatic laceration, fibrous fistulous tract, and surrounding adhesions. The diaphragm was intact, with no bile leakage or biloma observed. Complete removal was confirmed intraoperatively, with no residual FB identified.

**Table 1:** Perioperative laboratory results.

Parameter (unit)	Normal range	18 December 2025
Hb (g/dl)	11.5–16.0	12.5
WBC (cell/μl)	4000–11 000	7200
Diff count (%):		
Eosinophil	1–2	5
Basophil	0–1	
Neutrophil	54–62	53
Lymphocyte	25–33	37
Monocyte	4–10	5
Platelet count (x10^3^ cell/μl)	150–450	296
PCV (%)	35–47	37.4
Erythrocyte sedimentation rate (mm/h)	0–20	7
Random blood glucose (mg/dl)	<125	86
Ureum (mg/dl)	15–45	32
Creatinine (mg/dl)	0.7–1.4	0.82
Bleeding time (min)	1–7′	3′0″
Clotting time (min)	3–10′	6′30″

The postoperative course was uneventful, and she was discharged on postoperative Day 4. She remained asymptomatic at 1-month follow-up, with no complications.

## Discussion

Intrahepatic FBs is a rare cause of delayed hepatic pathology and often associated with liver abscess formation [5]. Diagnosis is frequently delayed because early symptoms are nonspecific and a clear history of FB ingestion or penetrating injury is often absent, leading to misdiagnosis as cryptogenic liver abscess [2]. This case follows this pattern but is distinguished by its traumatic origin and thoracic-dominant presentation.

Most reported intrahepatic FBs result from migration following silent GI perforation by ingested sharp objects, including fish bones, sewing needles, metallic pins, and toothpicks [6]. Traumatic penetration is a less common but important mechanism, particularly in thoracoabdominal trauma involving glass or metallic fragments, and may be overlooked when early symptoms are mild or attributed to musculoskeletal causes [1, 3].

Delayed presentation is common in reported cases, with symptoms often emerging weeks to months after the inciting event [1]. In this case, the 10-month delay likely reflected the absence of early abdominal symptoms and an oblique thoracoabdominal trajectory, with callus formation at the left sixth rib supporting a healed penetrating mechanism that masked underlying visceral injury. This underscores the importance of applying Advanced Trauma Life Support (ATLS) principles in thoracoabdominal trauma, as careful attention during the exposure phase may reveal subtle entry wounds or penetration pathways and reduce diagnostic delay [7].

A distinctive aspect of this case was the transcostal trajectory, with the FB traversing the sixth intercostal space before penetrating segment III of the left hepatic lobe. Intrahepatic FBs more commonly involve in the left hepatic lobe, likely due to its close anatomical proximity to the stomach and duodenum, consistent with previous reports [5, 8]. As a result, chest wall pain and a palpable thoracic mass predominated, masking the underlying hepatic injury. Failure to identify and remove an FB may lead to persistent inflammation, recurrent infection, or systemic sepsis [5, 9]. Antibiotic therapy or abscess drainage alone is insufficient when the FB remains *in situ* [9]. In this patient, exploratory laparotomy was performed to exclude GI injury before FB extraction.

In summary, intrahepatic FB should be considered in delayed or atypical presentations after thoracoabdominal trauma, as healed rib fractures may obscure visceral injury. Strict adherence to ATLS principles, particularly the Exposure phase, with timely imaging and definitive FB removal, is crucial to prevent liver abscess and other serious complications.

## Conclusion

Intrahepatic FB should be considered in delayed or atypical presentations following thoracoabdominal trauma, as subtle penetrating mechanisms, thoracic-dominant symptoms, and healed rib fractures may obscure underlying hepatic injury. Adherence to ATLS principles, particularly during the Exposure phase, appropriate imaging, and definitive FB removal are essential for timely diagnosis and prevention of serious complications.
